# Tailoring agarose fluid gels for use in suspension bath bioprinting and culture of spheroid-based bioinks

**DOI:** 10.1088/1758-5090/ae0aff

**Published:** 2025-10-10

**Authors:** Megan E Cooke, Nikolas Di Caprio, Jason Killgore, Jason A Burdick

**Affiliations:** 1 BioFrontiers Institute, University of Colorado Boulder, Boulder, CO 80303, United States of America; 2 Department of Bioengineering, University of Pennsylvania, Philadelphia, PA 19104, United States of America; 3 Applied Chemicals and Materials Division, National Institute of Standards and Technology, Boulder, CO 80305, United States of America; 4 Department of Chemical and Biological Engineering, University of Colorado Boulder, Boulder, CO 80303, United States of America

**Keywords:** suspension bath, spheroids, bioprinting, bioinks, agarose fluid gel

## Abstract

Suspension bath bioprinting, whereby bioinks are extruded into a yield stress bath with rapid recovery from shearing, has enabled the printing of low viscosity bioinks into constructs with high geometric complexity. Previous studies have often relied upon external stabilisation of the suspension bath (e.g. collagen) in order to culture soft materials without loss of printed structure. Here, we report a systematic investigation of suspension bath properties that support the printing, fusion, and culture of spheroid-based bioinks without added stabilisation. Specifically, agarose fluid gels of varied polymer concentrations and dilutions were produced and characterised morphologically and rheologically. Juvenile bovine chondrocytes or mesenchymal stromal cells (MSCs) were formed into spheroids of ∼150 *µ*m in diameter and investigated within agarose suspension baths either for their fusion in hanging drop cultures or as jammed bioinks. MSC spheroids were also printed when mixed with hydrogel microparticles to demonstrate additional versatility to the approach. Suspension baths of lower polymer concentrations and increased dilution enabled faster spheroid fusion; however, the most heavily diluted suspension bath was unable to maintain print fidelity. Other formulations supported the printing, fusion, and culture of spheroid-based inks, either as simple lines or more complex patterns. These findings help to inform the design of suspension baths for bioprinting and culture.

## 1. Introduction

Technological developments in the 3D printing of soft and biological materials have accelerated over the last 20 yrs and as such, bioprinting is now a commonly used technique in tissue engineering laboratories worldwide. Concurrently, a range of bioinks—printable formulations containing cells either alone or with materials as carriers—have been developed for various applications [1]. To ensure good printability with extrusion printing techniques, bioinks must fulfil certain rheological criteria. Specifically, they must exhibit shear-thinning behaviour, with clearly defined yield stresses for controlled extrusion, and show very little thixotropic behaviour to ensure quick recovery of solid-like properties to enable printed shape fidelity upon deposition [2]. These requirements may be restrictive and often encourage the use of higher viscosity bioinks that reduce cell viability, as increased shear stresses are required for bioink extrusion [3].

One solution for this arose around a decade ago with the emergence of suspension bath bioprinting. In this bioprinting modality, bioinks are extruded into a suspension bath of a yield stress material that exhibits rapid shear recovery [2]. A wide variety of suspension baths have been developed, which range from granular media to viscous liquids and include molecular assemblies such as guest-host systems and even nano-clay suspensions [4–6]. Surface tension and molecular interactions between the bioink and suspension bath enable the printing of constructs with high geometric complexity, even with materials that do not fulfil the rheological criteria for directly extruded bioinks. In most cases, post-extrusion crosslinking of the bioink or suspension bath are applied to stabilise the structure, such as through photo-, ionic-, or temperature-based crosslinking mechanisms [4, 7–9]. In some systems (collectively known as FRESH), the suspension bath is ‘reversed’, for example by heating of a gelatin suspension bath above its gelation temperature to release the printed part, while others gently wash away the suspension bath to release the part. The inverse is also possible, where the suspension bath is crosslinked and maintained, while the ink is removed, which is useful to create pores, channels and other features [10, 11].

While suspension bath bioprinting has dramatically enhanced the printability of soft and biological materials, bioinks containing hydrogels may impede tissue growth by physically blocking the deposition of neotissue. Additionally, the use of hydrogel-based bioinks containing dispersed cells often fail to achieve the cell densities or cell–cell interactions that are needed to recapitulate various tissue types (e.g. cartilage, cardiac) [12, 13]. One solution to avoid this issue has been to take inspiration from embryonic tissue development and use cell-only bioinks [14, 15]. Notably, suspension bath bioprinting has enabled cell-only bioprinting strategies such as with the use of cell slurries. For example, work from the Alsberg and Angelini groups have shown the extrusion of cell-only bioink slurries that condense post-extrusion after subsequent crosslinking of the suspension bath [16, 17].

Beyond cell slurries, cell spheroids also hold many advantages when compared to cells distributed throughout hydrogels, primarily in that high cell densities and direct cell–cell contacts are inherent to the system [18]. Cell spheroids form when cells are placed in hanging droplet or U-well cultures and dynamically assemble into spheres to minimise their surface energy, known as cell condensation [18]. When placed in contact with other spheroids, fusion occurs, which allows the formation of larger structures [19, 20]. Cell spheroids have been used as bioinks in biofabrication for several decades, but their use has grown in recent years as the technology has improved to control their deposition and use as tissue building blocks [21–24]. As one example, the Kenzan method utilises solid needle-like supports for the precise deposition of cell spheroids, which are then allowed to fuse [25, 26]. The tissues produced have good biochemical and mechanical properties [27], but the technology for this technique is expensive and the production rates are relatively low throughput.

Suspension bath bioprinting enables the gentle deposition of spheroid-only bioinks into 3D structures. For example, aspiration-assisted freeform bioprinting has been developed as an effective strategy to spatially deposit individual spheroids within guest-host or granular suspension baths to allow their fusion into hierarchical tissue models, but this approach suffers limitations of low throughput [24, 28, 29]. Inversely, others have demonstrated the use of jammed spheroids (or organoids) with a collagen or basement membrane interstitial matrix as a yield-stress suspension bath, where a sacrificial material can be printed to form ‘vascular’ channels within the extremely high cell dense solid structure [6, 17, 30, 31]. However, the use of an interstitial matrix can reduce spheroid fusion. Regardless of the strategy, it still remains a challenge to control the fusion and growth of these bioinks over time, which motivates the need to engineer suspension baths that guide, modulate, and maintain spheroid fusion and culture without external stabilisation.

**Figure 1.**
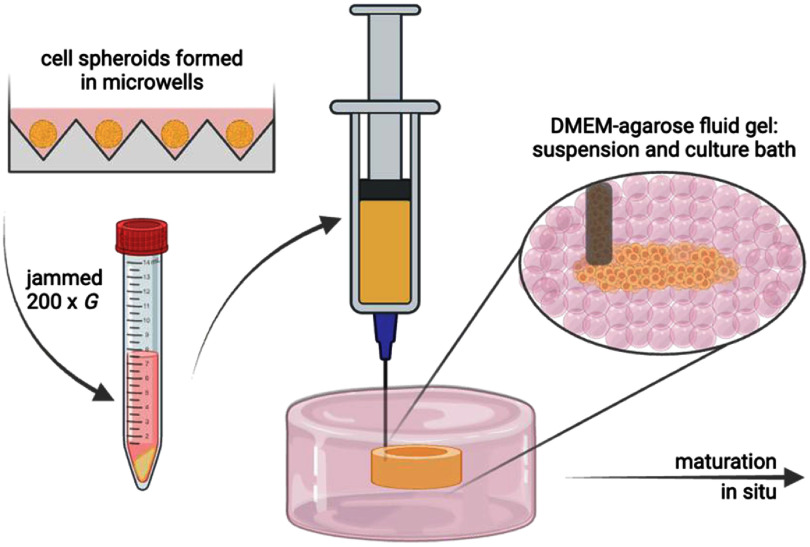
Schematic illustration of the suspension bath print, fuse, and culture system. Top left: spheroids (e.g. chondrocytes, MSCs) are formed in microwells before being collected and jammed by centrifugation into a paste-like bioink. The jammed spheroid-based bioink is then loaded into a syringe and printed into an agarose fluid gel suspension bath with controlled biophysical properties. No additional stabilisation is applied and the spheroids are allowed to fuse and mature as a tissue *in situ*.

With these considerations in mind, the goal of this study is to advance suspension bath bioprinting for the controlled patterning, fusion, and culture of spheroid-based bioinks without external stabilisation. To accomplish this, we formed spheroids within microwells from chondrocytes as well as mesenchymal stromal cells (MSCs) and assessed features such as their fusion, which then informed the design of jammed spheroid-based bioinks for printing, fusion, and culture within suspension baths (figure 1). There are numerous suspension baths that can be used in such a study, from molecular assemblies through to granular media, each with their own challenges and advantages [32]. Here, we selected agarose fluid gel suspension baths due to (i) their simplicity in fabrication that can be used across any laboratory, (ii) their high cytocompatibility when used with cell-based bioinks, and (iii) their use without the need for any additional crosslinking to support spheroid fusion over time in culture [33]. Agarose fluid gel suspension baths were formed in Dulbecco’s Modified Eagles Medium (DMEM) and a systematic study was performed on their use across varied polymer concentrations and bath dilutions to understand how these properties influence the printing, fusion and culture of spheroid-based bioinks.

## 2. Methods

### 2.1. Materials

All chemicals including agarose were purchased from Millipore Sigma and all cell culture reagents and staining solutions were purchased from Life Technologies (ThermoFisher).

### 2.2. Preparation and analysis of agarose suspension baths

Agarose suspension baths were produced as previously described in the preparation of agarose fluid gels [7]. Briefly, 1, 0.75 or 0.5 w/v% high-melt agarose (Millipore Sigma) was added to unsupplemented DMEM (DMEM, Gibco). This was then autoclaved for sterilisation before being sheared at 850 rpm on a magnetic stirrer until cooled to room temperature (20 °C). Chondrogenic supplements (1% Penicillin Streptomycin (PS), 1% non-essential amino acids, 1% Insulin Transferrin Selenium, 50 *µ*g ml^−1^ ascorbic acid, 100 nm dexamethasone, 10 ng ml^−1^ transforming growth factor-*β*) were then added. For ‘diluted’ baths, a 1% agarose fluid gel was diluted with chondrogenic media to achieve 0.75% or 0.5% polymer concentration (denoted 0.75% D and 0.5% D).

For analysis of agarose particle morphology, solutions were prepared in phosphate buffered saline (PBS) with 0.01 wt% FITC–dextran (700 kDa, Millipore Sigma) prior to shearing. FITC–dextran containing agarose particles were dispersed into warm 2% gelatin (from porcine skin, Millipore Sigma) and allowed to cool before imaging under confocal microscopy (Nikon AXR). 3D volumes were reconstructed for visualisation of particle morphology (Nikon elements).

The mechanical properties of agarose suspension baths and jammed cell spheroids were quantified using both rotational and small amplitude oscillatory rheology (Discovery HR-20, TA Instruments). Flow sweeps were performed with increasing shear rates from 0.003 to 500 s^−1^, strain-controlled amplitude sweeps were performed between 0.01% and 500% at 1 Hz, and 5-step thixotropic measurements were performed between oscillation strains of 0.1% and 200% at 1 Hz. Fluid agarose suspension baths were measured using 20 mm parallel plates with a 1 mm gap height.

### 2.3. Diffusion permeability assay

Diffusion permeability assays were performed within a custom polydimethylsiloxane (PDMS) chamber. PDMS and crosslinker (10:1 ratio) were cast between two glass coverslips with a gap height of 2 mm at 80 °C, cut to create ∼1-2 mm channels with lengths ∼20 mm, and plasma bonded to large glass coverslips. The agarose suspension bath was injected into the channels to fill ∼15 mm of the length, immediately sealed with a coverslip, and placed within a 15 cm petri dish with wet Kim wipes around the periphery to reduce evaporation. A calcein solution (MW: 622.55 g mol^−1^, 10 *µ*m) was injected into ∼5 mm of the remaining channel, immediately sealed with a coverslip, and imaged. Dye diffusion was imaged on a stereoscope (Nikon SMZ18) with 488 nm filter cube every 4 min for 2 h. Diffusion permeability was then calculated through mass flux using the equation: *J* = *P*_D_(*c*_sink_-*c*_agarose_), as previously described [34]. Briefly, the initial fluorescence intensity, sink volume, and cross-sectional area were used to determine the concentration of the sink (*c*_sink_). The fluorescence intensity and area were used to determine the concentration of the agarose (*c*_agarose_) and was plotted against each time interval and fit to a linear profile, whose slope was used to calculate the overall diffusion permeability (*P*_D_) of calcein within the agarose suspension bath. 3 replicates were performed.

### 2.4. Spheroid production and characterisation

Chondrocytes were isolated from juvenile (10–14 d) bovine knee joints (purchased from Research87). Briefly, cartilage was removed from the tibial plateau and femoral condyles with a scalpel before being minced into 3-4 mm^2^ pieces. They were then digested in 1.5 mg ml^−1^ type II collagenase (LifeTechnologies) in DMEM for 16 h at 37 °C before the solution was strained through a 70 *µ*m cell strainer (PluriSelect). Chondrocytes were then plated onto adherent tissue culture plastic and expanded in monolayer culture in DMEM with 10% fetal bovine serum (FBS) and 1% PS for up to 4 passages. MSCs were obtained from adult Yucatan mini-pig femoral bone marrow using standard plastic adherence procedures. MSCs were cultured in *α*-Modified Eagle’s Medium (*α*-MEM), 10% FBS, 1% penicillin/streptomycin and 1 ng ml^−1^ betafibroblast growth factor. MSCs were passaged up to P3 using 0.05% Trypsin/EDTA and neutralised with equal parts *α*-MEM + 10% FBS.

Agarose (2 wt%) microwells were cast using a PDMS stamp in 6-well plates. PDMS stamps were created by casting PDMS into AggreWell 400 (StemCell Technologies), degassing and curing at 80 °C. Cells were seeded into each well and allowed to settle and aggregate, resulting in ∼1250 cells per spheroid. Spheroid compaction was then quantified by imaging spheroids in culture under widefield microscopy (Nikon Ti2) over a period of 7 d and measuring their mid-point cross-sectional area, as well as aspect ratio and circularity (ImageJ).

### 2.5. Atomic force microscopy (AFM)

An AFM (Cypher, Oxford Instruments, UK) was used to probe the local modulus of spheroids after 1, 3 and 7 d of pre-culture. Spheroids were aspirated from their microwells and deposited onto aminosilane-functionalised glass coverslips for 90 min prior to testing to ensure slight adherence to the substrate. Before testing, culture media was removed and replaced with PBS. Measurements were performed in solution, with a cantilever with 0.35 N m^−1^ spring constant with a 12 *µ*m borosilicate sphere attached as the cantilever tip. Spheroids were identified with the AFM’s optical microscope and the tip was positioned at the apex of the spheroid. A maximum force of 10 nN was applied, and measurements were performed in a 6 × 6 array across a 10 *µ*m × 10 *µ*m area. Force versus displacement curves were fit to a Hertz contact mechanics model to determine modulus. Measurements were repeated on at least 5 spheroids per incubation condition.

To visualise the spheroid attachment to aminosilane coated glass, spheroids were incubated with cell mask (LifeTechnologies) for 30 min and washed in fresh media prior to attachment for 90 min. They were then fixed with 4% paraformaldehyde (PFA) for 30 min and then imaged using confocal microscopy (Nikon AXR).

### 2.6. Immunohistochemistry

Spheroids were aspirated from agarose wells and fixed for 30 min in 4% PFA (Electron Microscopy Sciences) in PBS before washing with PBS 3 times for 5 min each, centrifuging between washes. Spheroids were then permeabilised for 20 min in 2% Triton-X before blocking for 1 h in PBS with 10% donkey serum and 0.3% Triton X. Spheroids were incubated with primary antibodies in vehicle (PBS with 10% donkey serum) overnight at 4 °C. Primary antibodies with dilutions used were: aggrecan (MA3-16 888, ThermoFisher, 1:50); Collagen Type II (ab34712, abcam, 1:50); N-Cadherin (C3865, Millipore Sigma, 1:100); tenascin-C (MA5-16 086, ThermoFisher, 1:50). After overnight incubation, samples were washed 3 times for 5 min in ice cold vehicle before secondary antibodies (donkey anti-rabbit 555 and goat anti-mouse 488, ThermoFisher) at 1:200 dilutions were added and incubated for 1 h in the dark. DAPI was added for the last 5 min at 1:5000 dilution. Samples were washed 3 times in vehicle before clearing in RapiClear 1.52 (SunJin Lab) for 2 h. They were then imaged using confocal microscopy (Nikon AXR).

### 2.7. Hanging droplet culture

20 *µ*l droplets of media or suspension bath were pipetted onto the top cover of a 35 mm dish and two spheroids were placed before being inverted for the duration of the experiment. The 35 mm dish was partially filled with PBS to prevent dehydration of the droplets during culture. Spheroids were imaged on a widefield microscope (Nikon Ti2) at a series of timepoints, capturing the mid-plane of the spheroid pairs. Fusion was quantified by the liquid drop model [35], which compares the radius of the initially seeded spheroids (*R*_0_) and the radius of the neck between spheroids (*r*_0_).

### 2.8. Jamming spheroids into a bioink

To form a printable paste-like bioink, cell spheroids were aspirated from their microwells and centrifuged. Different rates of centrifugation (200, 1000, 2000 × g) were used to determine the impact of centrifugation on cell viability after 1, 3, and 7 d of spheroid compaction. Spheroids were aspirated from their wells, centrifuged for 3 min at the above rates and then immediately stained with calcein-AM and ethidium homodimer-1 (4 *µ*m and 2 *µ*m respectively, LifeTechnologies). After 10 min, staining solutions were removed, PBS added, and spheroids were imaged using confocal microscopy (Nikon AXR).

Rheology was performed on jammed spheroids after 1, 3 and 7 d of pre-culture. Spheroids were aspirated from agarose microwells and jammed for 3 min at 200 × g. The supernatant was aspirated, and spheroids were transferred using a spatula to a sandblasted lower geometry. Shear rate sweeps from 0.005 to 50 s^−1^ and oscillatory strain sweeps from 0.05%–500% at 1 Hz were performed using 8 mm sandblasted parallel plates with a gap height of 750 *μ*m (Discovery HR-20, TA Instruments).

### 2.9. Spheroid-based printing and culture

All printing was performed using a modified Prusa Mini+ inside a biosafety cabinet to maintain sterility. Spheroids were jammed at 200 × g for 3 min and then loaded into 100 *μ*l Hamilton syringes with 30 G needles. Agarose bath material was deposited into individual wells of a 12 well plate and lines of 5 mm in length were deposited. Between each line, the needle was retracted from the bath to prevent smearing of spheroid ink material. Following printing, lines were immediately imaged and then maintained in their baths for 7 d, with each bath replenished with self-bath material after 2 and 5 d. Image analysis was performed on printed filaments by removing the background of the bath before performing shape and edge analyses using ImageJ. The ‘Analyze Stripes’ macro was used to quantify root mean square roughness (Rq) of each printed filament long edge (two edges per filament). Specifically, the vertical deviations from the mean line of the edge were quantified.

### 2.10. Macromer synthesis, characterisation, and microgel fabrication

Norbornene-modified HA (NorHA) was synthesised via organic synthesis with the tetrabutylammonium form of HA [36]. Specifically, sodium HA (Lifecore, 66 kDa) was dissolved in DI water and mixed with Dowex 50 W × 200 proton exchange resin for 2 h, titrated to pH ∼7.02–7.05 with tetrabutylammonium hydroxide (TBA, 0.2 m), frozen at −80 °C and lyophilised to form HA-TBA. HA-TBA was then modified with norbornene functional groups through benzotriazole-1-yl-oxy-tris-(dimethylamino)-phosphonium hexafluorophosphate (BOP) coupling. The synthesised HA macromer was purified and then characterised with ^1^H NMR (*δ* ≈ 5.8–6.2, 2 H) and the extent of modification was calculated as 19%.

NorHA microgels were fabricated via water-in-oil batch emulsions as previously described [37]. Emulsions were carried out in a 100 ml glass beaker (Ø:4.5 cm, H:6.5 cm) with a Teflon coated stir bar (L:4 cm, W: 1 cm) and using an 80:1 oil:macromer solution volume ratio stabilised with 2% Span 80. A macromer solution was prepared at a final concentration of 3 wt% in PBS, with photoinitiator (I2959, 0.05 wt% final) and crosslinker (dithiothreitol, DTT, 10 mm). The macromer solution was added dropwise into the spinning oil bath (RPM = 350), allowed to equilibrate for 30 s, and irradiated with UV light (20 mW cm^−2^) for 15 min. Microgels were collected and washed with 1% tween 20 and subsequent washes (5 washes) with 1X PBS. A filtration step was added to remove any large microgels with Ø > 200 *µ*m.

### 2.11. Spheroid and hydrogel microparticle printing, culture, and removal

MSC spheroids were disrupted from microwells via mixing with pipette and transferred to a conical tube on ice. Spheroid suspensions were centrifuged at 300 × g for 20 s and microgels were centrifuged at 15 000 × g for 5 min to determine total volume. 2.5 mm DTT and 0.05% LAP photoinitiator were added to the microgels to allow for interparticle crosslinking, vortexed, and centrifuged at 15 000 × g for 5 min. The spheroids and microgels were added together at 35:65 microgel: spheroid volume ratio, manually mixed with a spatula, centrifuged for 20 s at 300 × g, and loaded into a 250 *µ*l Hamilton syringe with a 22 G blunt tipped needle to create the spheroid and hydrogel microparticle bioinks. Before printing, Hamilton syringes were capped with a luer-lock stopper and centrifuged at 500 × g for 30 s to remove any air bubbles that formed during loading. 5 ml of 0.75% agarose particle suspension bath was prepared with chondrogenic supplements and pipetted evenly within each well of a 4-well rectangular tissue culture plate. Initial filament printing was conducted using NorHA and spheroid inks at a length of 4, 8, 12 mm and cultured up to 7 d within agarose suspension baths. The needle speed was set to 1.7 mm s^−1^ while the flow rate was set to 0.30 mm s^−1^ while using a 22 G needle. Filaments were imaged immediately using a fluorescent widefield microscope (Nikon) and length was quantified in FIJI at end-to-end distance manually. 6 mm NorHA rings were printed using identical printing parameters apart from printing 2 layers to form the cylindrical object. The cylinders were printed and then cultured for 7 d in agarose bath with chondrogenic supplements at 37 °C. Ring thickness was manually measured and an average of 4 measurements around the ring structure were determined at each timepoint in FIJI.

### 2.12. Statistics

Statistical analyses were performed using GraphPad Prism v10. All data are plotted as mean ±SD. One-way and 2-way ANOVAs were performed as reported in figure captions with Tukey’s post-hoc testing for comparisons within timepoints and *p* < 0.05 denoting statistical significance.

## 3. Results and discussion

### 3.1. Characterisation of agarose fluid gels

**Figure 2.**
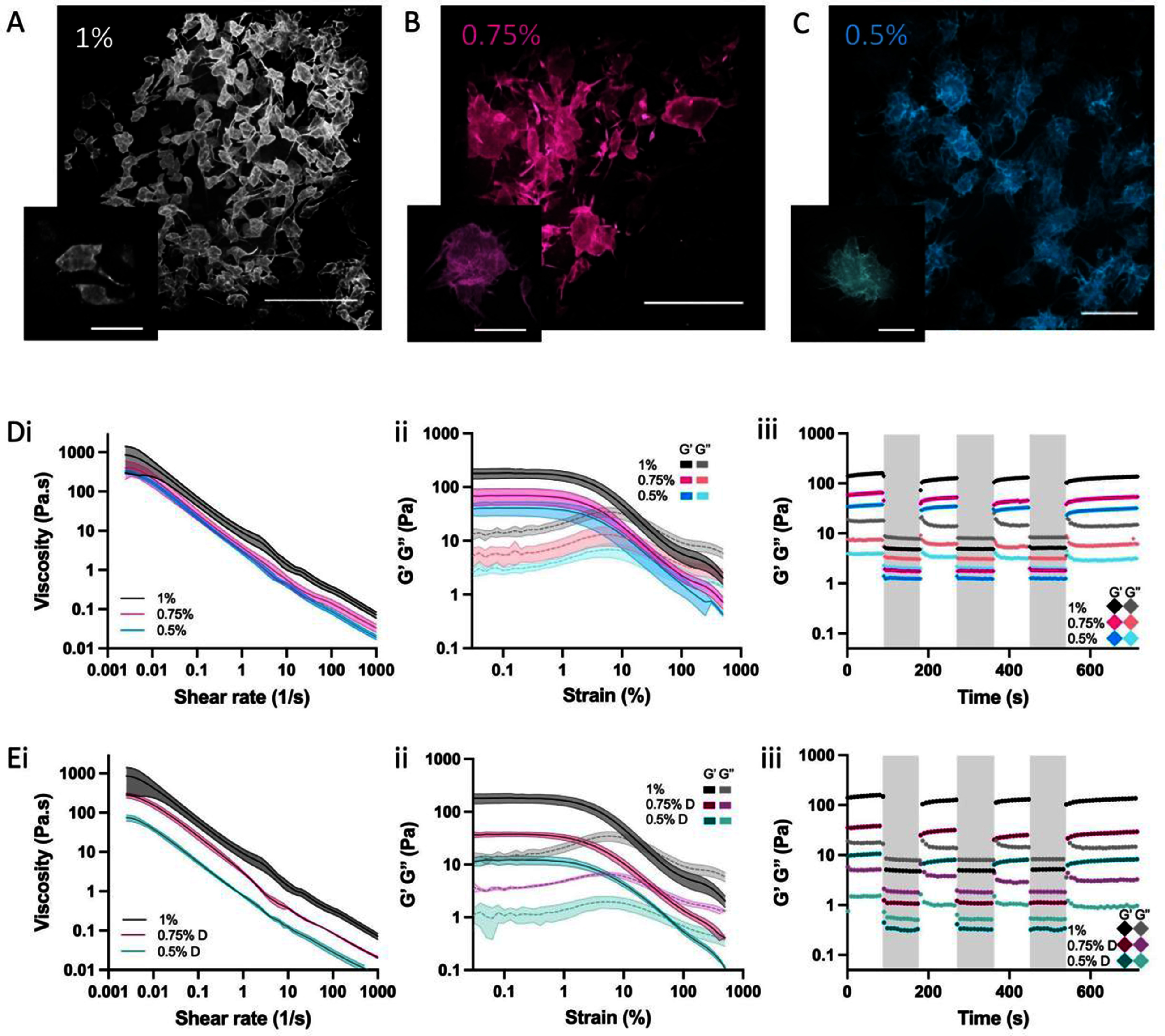
Morphological and rheological characterisation of agarose fluid gels. (A)–(C) Confocal microscopy of agarose fluid gels (1%, 0.75%, 0.5%) diluted to visualise individual particles (Scale Bar: 500 *µ*m), insets show individual particles (Scale Bar: 250 *µ*m). (D)–(E) i: Flow sweep, ii: oscillatory strain sweep, and iii: 5-step recovery profile of (D) ‘as-made’ (1%, 0.75%, 0.5%) or (E) diluted (1% alone or diluted to 0.75% (noted as 0.75% D) or 0.5% (noted as 0.5% D)) agarose fluid gels. *n* = 3 samples for all rheological experiments.

Agarose fluid gels were selected as simple to fabricate suspension baths, where features such as rheological properties can be readily modified for use in 3D printing (33). As suspension bath rheology is essential for printability, we first sought to better understand features of the agarose fluid gels across a range of formulations, where features such as polymer concentration (1%, 0.75%, 0.5%) and dilution (dilution of 1% fluid gel to 0.75% or 0.5%) were altered. Agarose fluid gels were fabricated through the shearing of agarose during gelation, which results in high concentrations of agarose particles. When formed with the addition of FITC–dextran, diluted solutions enabled the visualisation of individual particles via confocal microscopy (figures 2(A)–(C)). With decreasing polymer concentration, the individual particles (ranging from ∼150–300 *µ*m) were larger and contained more projections, which is consistent with previous observations (33). It is important to note that the agarose fluid gels do not contain any additional interstitial matrix between particles, beyond agarose polymers leftover from the gelation process, which is different from many other granular suspension baths (35). To ensure that nutrients can diffuse throughout these agarose fluid gels to support spheroid-based inks for suspension bath 3D bioprinting, diffusion permeability studies were performed on 0.75% fluid gels using calcein (MW: 622.55 g mol^−1^) as a proxy for soluble nutrients (supplementary figures 1 (A)–(C)). The diffusion permeability (*P*_D_) of calcein through this agarose fluid gel concentration was 1.84 × 10^−6^ cm s^−1^, which is consistent with alternative hydrogels (i.e. collagen) that have supported high cell viability during culture [38].

In addition to particle morphology, it is important to understand how the various formulations behave rheologically. When tested with variations in polymer concentration during agarose fluid gel formation, the viscosity (figure 2(D-i)) and storage (G’) and loss (G’’) moduli (figure 2(D-ii)) increased with increasing polymer concentration. When the 1% agarose fluid gel was diluted to 0.75% and 0.5%, decreases in viscosity (figures 2(E-i)) and storage/loss moduli (figure 2(E-ii)) were observed. The differences in G’ at 1% strain were significant between the 1% ‘as-made’ bath and all other baths, and G’ was significantly decreased (*P* = 0.0257) in a 1% bath diluted to 0.5% compared to a 0.5% ‘as-made’ bath (supplementary figure 2(A)). The smaller particle size in the 1% ‘as-made’ bath as well as the higher polymer concentration may contribute to its higher viscosity and moduli due to close packing of the system compared to larger particles in other ‘as-made’ baths. The flow point, or critical strain, where G’ and G’’ cross-over was not significantly different between the formulations, except for the 0.5% diluted bath (supplementary figure 2(B)). This was the only bath that did not show clear yielding behaviour with only a more gradual reduction in G’ with increasing strain.

All baths showed rapid recovery of storage moduli to around 80% of the initial modulus after each period of high strain, which then increased over time (figures 2(D-iii) and (E-iii)). The 0.5% bath showed recovery to ∼96% of the initial modulus after the first high strain step and, in the 1%, 0.75%, 0.75% D and 0.5% D baths, the final (longer, 3 min) recovery step showed continued recovery to higher values (supplementary figures 2(C) and (D)). This may be an effect of the particle size and shape, in that during high strain regimes as a bulk, the larger particles undergo less deformation than the smaller ones. Additionally, differences were observed in mechanical properties of ‘as-made’ and diluted baths, for example a 70% reduction in G’ between a 0.5% ‘as-made’ and 0.5% diluted bath, which is important to note for use as a suspension bath. As these agarose fluid gels are intended to culture spheroid-based inks over long culture periods (i.e. 7 d), the mechanical stability and degradation rate of these fluid gels are important to consider. While agarose gels can be degradable via bacterial enzyme secretion, the cell types used here for printing (chondrocytes/MSCs) will not secrete enzymes that are able to degrade agarose and as a result, the mechanical properties of these fluid gels remain stable over the duration of the culture period [39].

### 3.2. Formation and characterisation of chondrocyte spheroids

**Figure 3.**
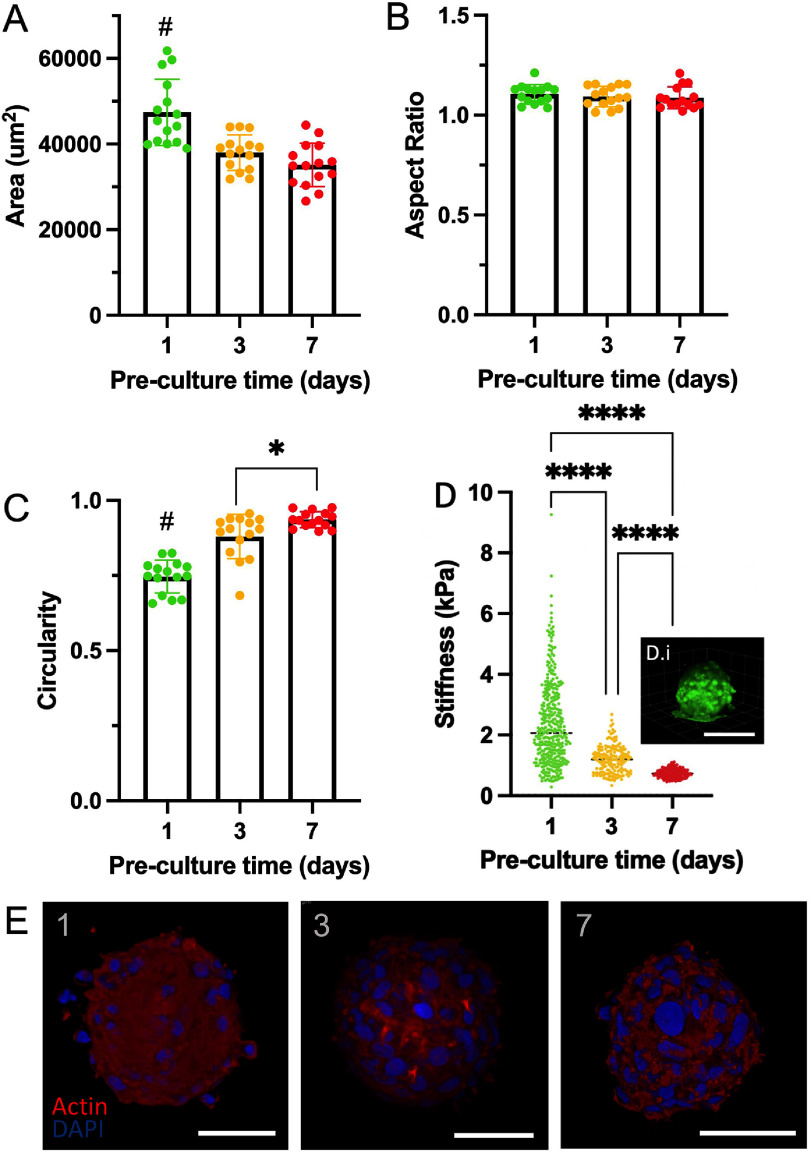
Formation and characterisation of chondrocyte spheroids. (A)–(C) morphological characterisation (area, aspect ratio, circularity) of spheroids over 7 d of culture (*n* = 15, One-way ANOVA for area: *P*< 0.0001, aspect ratio: ns, circularity *P* < 0.0001, # indicates statistical significance from all other groups,*P < 0.05). (D) AFM nano-indentation stiffness (*n* > 150 indentations) of spheroids after 1, 3, and 7 d of culture, inset (D.i) shows representative spheroid morphology during testing on aminosilane coated glass (Scale Bar: 50 *µ*m, ANOVA post-hoc testing ****P < 0.0001). (E) Actin and DAPI staining of chondrocyte spheroids after 1, 3, and 7 d of culture (Scale Bar: 100 *µ*m).

Chondrocytes were selected as a model cell due to the interest in spheroids for use in cartilage tissue engineering. Chondrocyte spheroids were readily formed through the deposition of chondrocyte suspensions onto agarose wells, where aggregation was observed approximately 6 h after cell seeding and sufficiently condensed after 24 h to allow aspiration from their microwells without disruption of their spherical shape. To better understand the features of individual chondrocyte spheroids, their properties over 7 d were monitored. Between 24 h and 7 d, spheroids were measured for size, shape, and circularity. There was large variability in cross-sectional area of spheroids at day 1, but this quickly reduced as the spheroids stabilised around 4 d to a diameter of ∼150 *µ*m (figure 3(A)). Across 7 d, spheroid aspect ratios were consistently around 1 with a narrow range, indicating that there was no spheroid elongation, cell outgrowth or adherence to the agarose microwells (figure 3(B)). However, there was a gradual trend towards increased circularity from 1 to 7 d, with no significant differences after 4 d, indicative of enhanced condensation of the spheroid (figure 3(C)). These features were expected and confirm the production of spherical spheroids for use in printing. Towards printing, the size of spheroids is important to dictate the resolution of printed structures, as the minimal feature will be spheroid size. It is important to note that spheroids of a wide range of sizes can be produced, based on features such as agarose well dimensions and cell concentration within each well [40].

To further characterise spheroid properties, AFM was used to understand their local mechanical properties. Spheroids were cultured on aminosilane coated coverslips for 90 min prior to testing to secure them for AFM. As seen in figures 3(D-i), there was a strong interaction with the substrate, but the top of the spheroid that was to be indented remained spherical. The spheroid was indented with a 12 *µ*m borosilicate sphere, which is much smaller than the size of an individual spheroid, ensuring that the local rather than overall properties of the spheroid were being tested. AFM results indicate that the mean stiffness of the spheroid reduced with culture time from 1 to 7 d (figure 3(D)). This variability is likely due to the presence of cells at the surface, as well as a disorganised matrix over time. An understanding of this surface is important, as this is the interface that is presented towards spheroid fusion.

To better understand the spheroid composition and surface properties, a range of markers were investigated immunohistochemically (figure 3(E)). At day 1, the actin network was diffuse, which then increased over days 3–7. This likely explains the reduction in stiffness across these culture times as the disorganised actin filaments at the spheroid surface on day 1 may have a higher stiffness than the dissipated stress of more organised actin at later time points [41]. The cell–cell interaction marker N-cadherin slightly increased at days 3 and 7 compared to day 1, indicating enhanced cell–cell interactions with culture (supplementary figure 3). Tenascin-C is also of interest as it is highly expressed during embryonic cartilage maturation and in the perichondrium up to four weeks after birth, matching the age of these juvenile bovine chondrocytes [42]. Tenascin-C staining was somewhat diffuse across time points, but particularly for later times (supplementary figure 3), which showed reduced surface stiffness as measured by AFM. Further, the native chondrogenic markers aggrecan and Collagen Type II were present and increased with pre-culture time (supplementary figure 3), which is expected with chondrocyte re-differentiation when cultured in 3D after de-differentiation in monolayer (38,39). Generally though, there was heterogeneity across the imaged spheroids.

### 3.3. Hanging droplet assessment of spheroid fusion in agarose suspension baths

**Figure 4.**
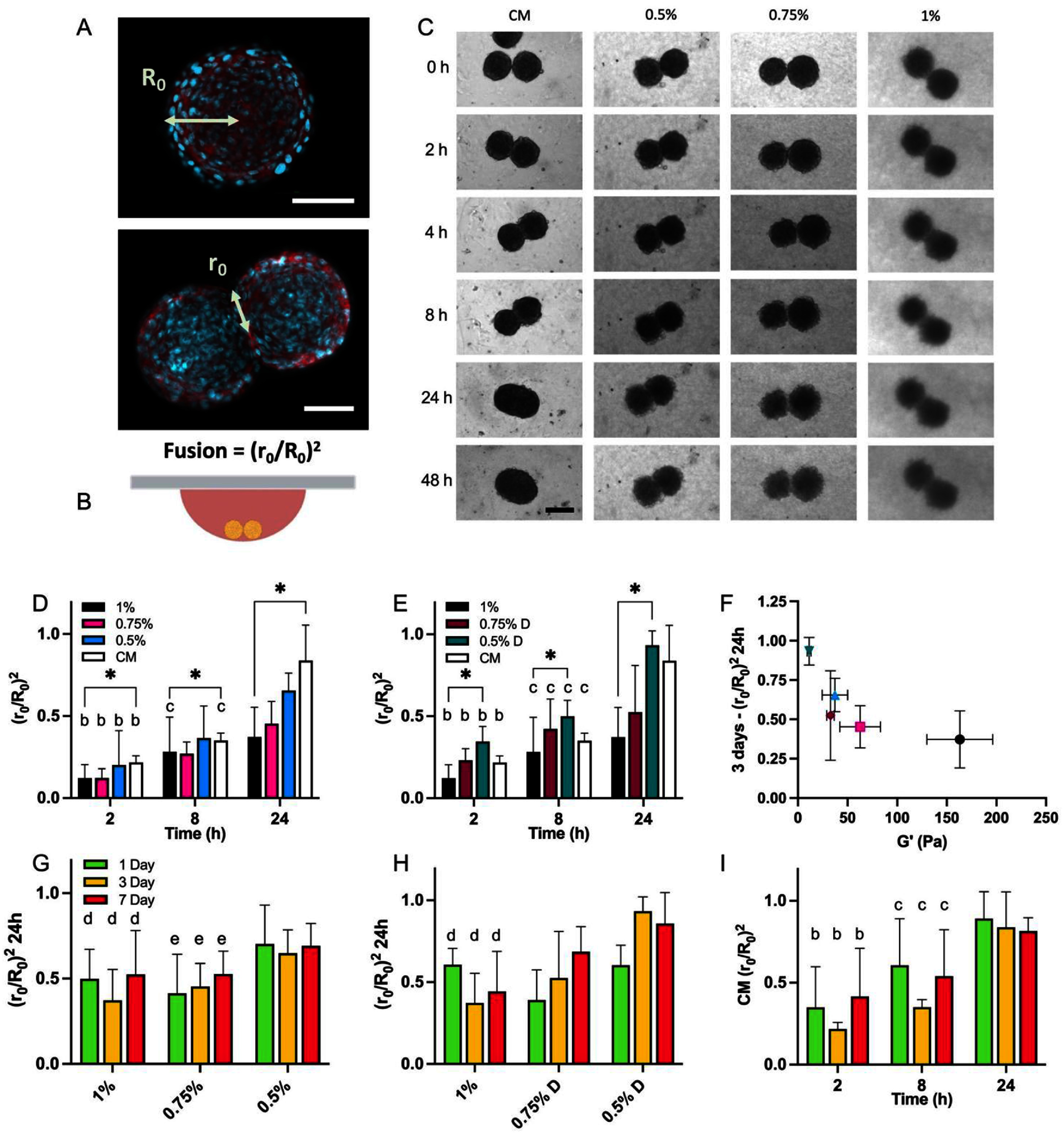
Analysis of hanging drop spheroid fusion experiments. (A) Spheroid fusion measurements (Red: actin, Cyan: nuclei, Scale Bar: 50 *µ*m), (B) hanging drop culture set-up, (C) representative examples of spheroid pairs in ‘as made’ agarose baths (Scale Bar: 100 *µ*m). Quantification of spheroid fusion after 3 d of preculture in (D) ‘as made’ baths and (E) diluted baths with 1% and culture media (CM) controls at 2, 4 and 24 h time points (*n* = 3-4 fusion measurements per time point for each bath formulation). (F) Relationship between storage modulus (G’) and fusion at 24 h in spheroids after 3 d of pre-culture. Comparison of pre-culture times on spheroid fusion in (G) ‘as made’ baths and (H) diluted baths, with (I) culture media controls over time (*n* = 3–7 fusion measurements per time point for each bath formulation). Results of post-hoc testing: *P < 0.05; b indicates significant difference between 2 h vs 24 h timepoint, *c* indicates significant difference between 8 h vs 24 h timepoint, *d* indicates significant difference between 1% and 0.5% (or 0.5% D) baths, e indicates significant difference between 0.75% and 0.5% baths.

As a first step to understanding spheroid fusion, pairs of spheroids were placed in hanging drop cultures of CM or the various formulations of agarose suspension baths [35]. The geometry of individual spheroids (*R*_0_) and their intersection with another spheroid (*r*_0_) during fusion can provide information of spheroid interactions over time (figure 4(A)). Specifically, chondrocyte spheroids were pre-cultured for 1, 3, or 7 d before being placed in the hanging drop cultures of either chondrogenic media or suspension baths (figure 4(B)) and imaged over a period of 48 h to assess their dynamic fusion (figure 4(C)). When comparing fusion over 24 h in ‘as-made’ baths (figure 4(D)), all spheroid pairs fused with the suspension baths over time, but to significantly lower extents than that of unrestricted fusion within CM controls. The fusion in CM was similar to previously reported data on cartilage spheroids doublets [43]. Similar trends were observed in the diluted baths (figure 4(E)).

Uncontrolled fusion is not desired, as this will prevent the maintenance of printed structures during the fusion and culturing process; thus, a fundamental understanding of fusion is needed. While hanging drop culture to assess spheroid fusion is a well-established technique, this, to our knowledge, is the first study of spheroid fusion in granular, semi-solid or viscoelastic material. Taken together, when comparing fusion at 24 h with the storage modulus of the bath (G’), a clear trend can be seen whereby increased modulus of the bath confers lower fusion (figure 4(F), supplementary figure 4). This indicates that with reduced bath modulus, there is less resistance to dynamic cell rearrangement in the formation of a single mass from two spheroids. Differences in particle sizes with changes in polymer concentration may also play a role in the accessibility of spheroid interfaces for fusion. The effect of pre-culture time on spheroid fusion was also investigated, but there were no significant differences observed between 1, 3 and 7 d of pre-culture time at the 24 h timepoint across any of the suspension baths (figures 4(G)–(I), supplementary figure 5). There were general trends towards 7 d of pre-culture leading to increased fusion in the 0.75% ‘as made’ bath and the diluted baths, which could be attributed to the softer surface of spheroids as measured by AFM (supplementary figures 5(B), (D), (E); figure 3(D)). The day 3 condition was often the lowest, which could also be linked to the increased tenascin-C expression and actin remodelling at this timepoint (supplementary figure 3; figure 3(E)); more deposition of boundary proteins could slow down the dynamic remodelling of the individual spheroids as tenascin-C has been shown to reduce cell spreading [44].

### 3.4. Rheology and viability of jammed bioink

**Figure 5.**
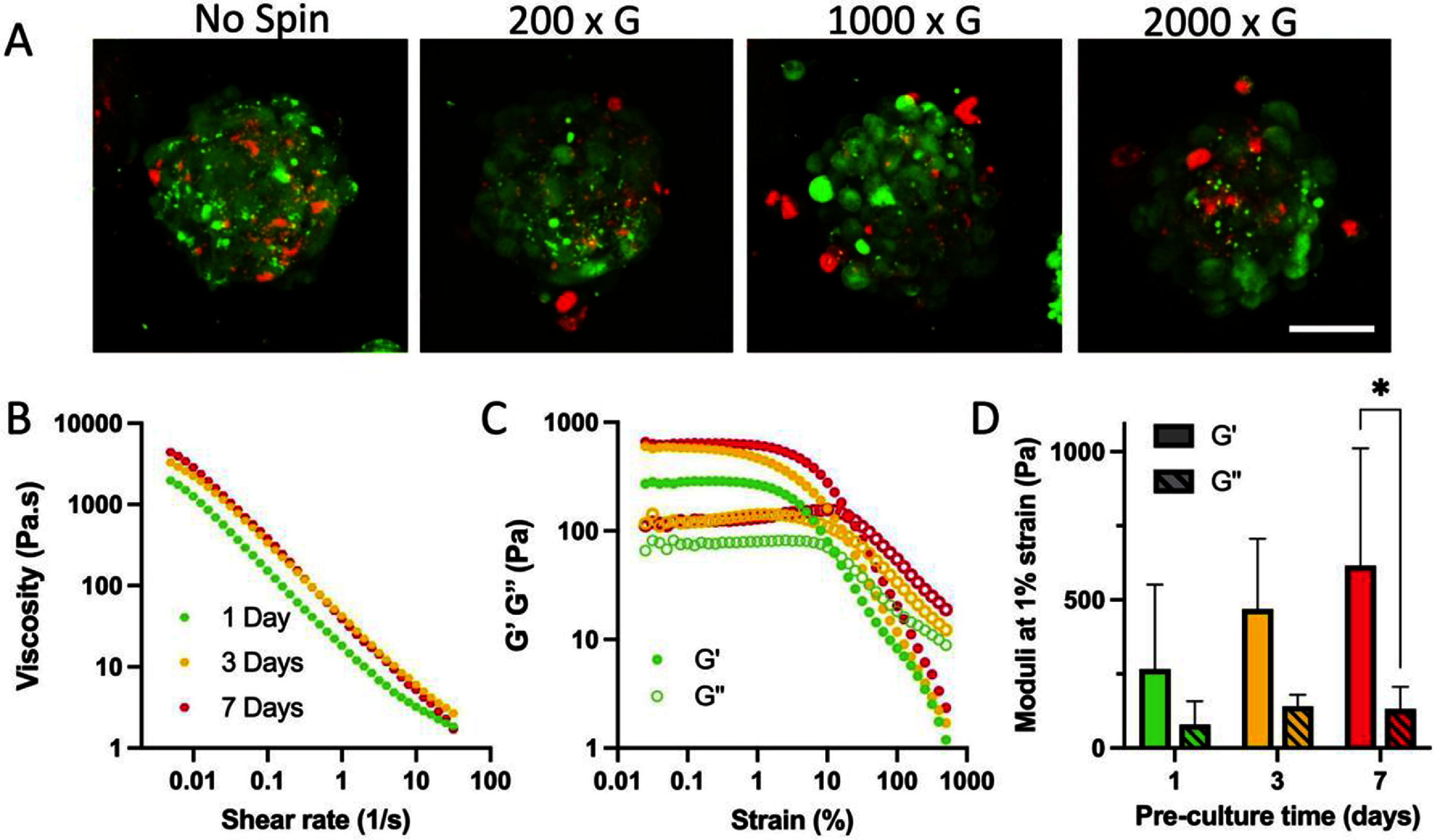
Jammed chondrocyte spheroids bioink characterisation. (A) Live-Dead staining of chondrocyte spheroids (3 d of pre-culture) jammed by centrifugation at different speeds (Scale Bar: 50 *µ*m, Live: Green, Dead: Red). (B) Flow sweeps, (C) oscillatory strain sweeps, and (D) moduli at 1% strain (*n* = 3) of spheroids of various pre-culture times jammed at 200 × g, post-hoc testing within pre-culture days: * *P* < 0.05.

To form a bioink for printing, chondrocyte spheroids were simply formed using microwells and then jammed by centrifugation. Features such as the viability of the cells within the spheroid-only bioink and the rheological properties are necessary for use in printing. To understand viability, various centrifugation speeds were used for jamming (figure 5(A)). There was a general observation that spheroid viability decreased with increasing centrifugation speeds. For instance, at higher speeds (1000 and 2000 × g), there were a lot of cells present at the base of the imaging chamber that had been sheared away from the spheroid during centrifugation. Direct quantification of the viability is challenging due to the dense nature of the cell aggregates, but based on these qualitative observations, the lower jamming speed (200 × g) was used in subsequent studies.

Similar to the agarose fluid gel rheological characterisation, jammed spheroid-only bioinks were characterised for features such as viscosity and yielding behaviour, which are important for use as an extrudable bioink. Flow sweeps confirmed that at all days of pre-culture, spheroids jammed at 200 × g exhibited shear-thinning properties (figure 5(B)), which will allow extrusion from the needle during printing. Further, all bioink formulations showed both linear and flowable regions with increasing oscillatory strain, as well as clear yielding behaviour (figure 5(C)), ensuring controlled extrusion. The storage moduli (G’) of jammed spheroids increased with pre-culture time but the trends were not significantly different (figure 5(D)). This may be attributed to the reduction in size and stiffness of spheroids with increasing pre-culture time, which enables closer packing into a denser bioink, whereas the overall heterogeneity of the spheroids and the packing results in diverse properties. For subsequent printing studies, spheroids of 3 d pre-culture were jammed at 200 × g.

### 3.5. Printing of jammed spheroid bioinks into agarose suspension baths

**Figure 6.**
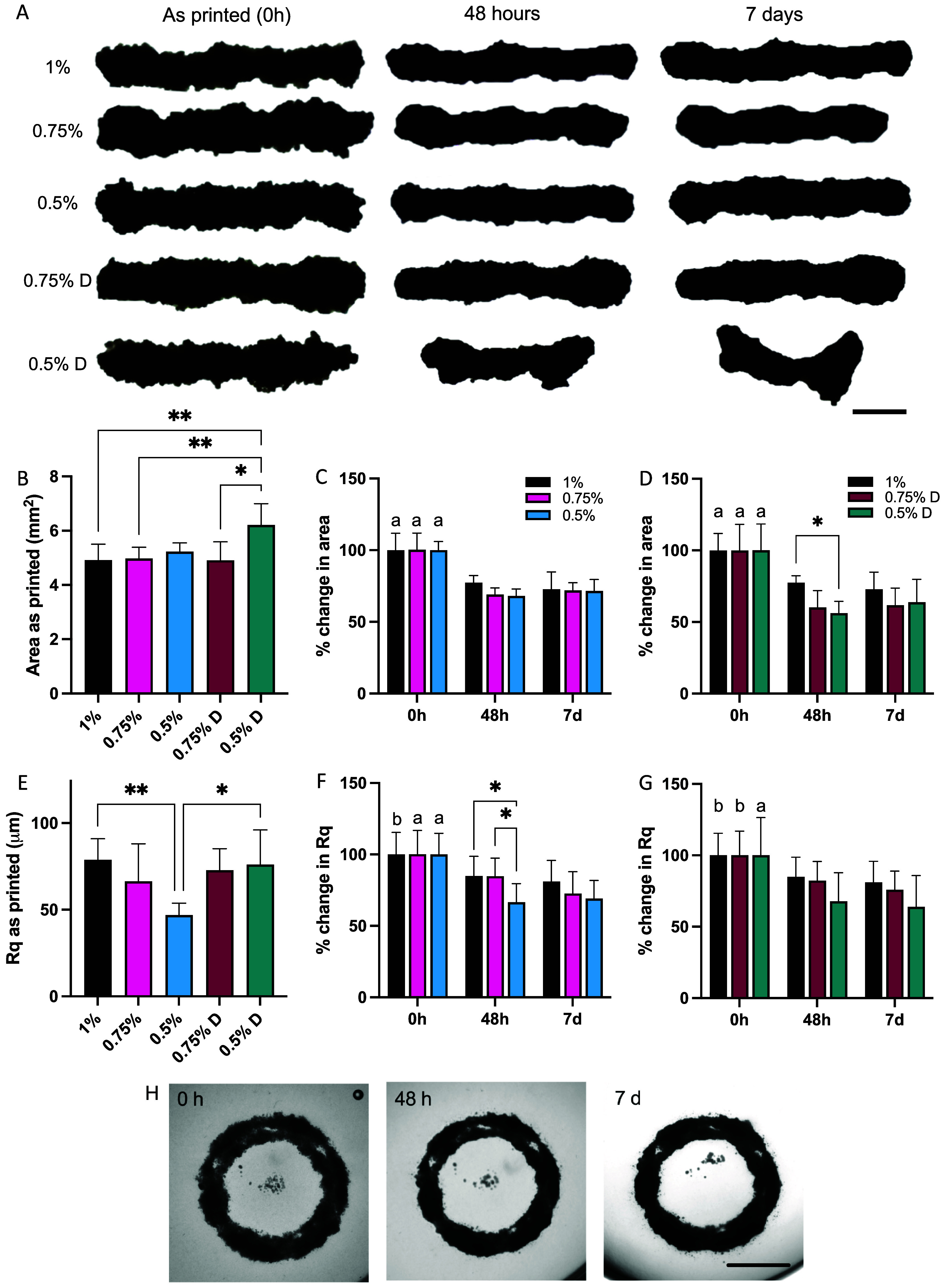
Printing of chondrocyte spheroid-only inks into agarose suspension baths. (A) Representative examples of printed filaments in each bath as printed, at 48 h and at 7 d of culture in the suspension baths (Scale Bar: 1 mm). Area of filaments (B) ‘as printed’ and over time in (C) ‘as made’ baths and (D) diluted baths. Rq of filament surface, (E) ‘as printed’ and over time in (F) ‘as made’ baths and (G) diluted baths. (H) Ring structures as printed and after 48 h and 7 d of culture within the suspension bath (Scale Bar: 3 mm). Results of post-hoc testing: * *P* < 0.05, ** *P* < 0.005; within baths, a indicates significant difference between 0 h vs 48 h and 7 d timepoint, b indicates significant difference between 0 h vs 7 d timepoint. *n* = 3–13 lines per formulation for quantification.

With a thorough characterisation of both the spheroid-only bioinks and the agarose suspension baths, it was possible to complete a systematic study of the printing of spheroid-only inks into suspension baths of varied properties (i.e. polymer concentration, bath dilution). One spheroid-only bioink formulation (3 d of chondrocyte spheroid pre-culture jammed at 200 × g) was successfully printed into each of the 5 agarose baths (figure 6(A)). As an initial step and for simplicity and quantification, simple lines were printed within the baths. ‘As-printed’ lines were largely successful across bath conditions; however, great care was needed to transfer the 0.5% diluted bath between the bioprinter in the biosafety cabinet and the microscope to ensure that the bath and print were not disrupted immediately after printing. In some cases, printed spheroids in this condition appeared completely diffuse after moving the culture plates. The ‘as-printed’ filaments were consistent in area across baths, except for the 0.5% diluted bath (figure 6(B)). This can also be seen in the difference in aspect ratio ‘as printed’, where the 0.5% diluted bath was significantly lower in aspect ratio than all other baths (supplementary figure 6). The lack of structure in this bath likely led to rapid diffusion of the spheroid inks into fluid pores between agarose particles when compared to the other baths that exhibited densely packed structures. When considering the roughness of the long edges of the ‘as printed’ filaments, the Rq (root mean square) value decreased from the 1–0.75–0.5% in ‘as-made’ suspension baths. The 0.5% bath showed significantly lower edge roughness when compared to the 1% bath (figure 6(E)), and this trend may be attributed to the larger agarose particle size. When the translating needle is closer to the size of the agarose particles, they are more effectively displaced, which may allow smoother deposition of the spheroid ink and hence lower Rq values. This trend does however oppose what was observed in the FRESH printing systems, between their first iteration using fragmented gelatin particles with diameters of around 65 *µ*m and second iterations of gelatin coacervates with diameters of 25 *µ*m [6]. Ultimately, the initial print pattern is very important in successful printing of inks.

Over 48 h post-printing, in all conditions, the printed filaments fused and began to contract (figures 6(A), (C) and (D)). This continued to the day 7 time point. In the 0.5% diluted bath, the printed filaments mostly stayed in one piece but became very deformed, likely due to the lack of confinement by the weak and very fluid suspension bath. When supplementing the suspension baths with fresh DMEM-agarose bath material, great care was needed for the diluted baths to ensure the filaments were not deformed simply by fluid flow of bath material. Significant differences in filament area were observed between baths at the 48 h timepoint, and the diluted baths generally showed larger decreases in area from the 1% control compared to the ‘as-made’ 0.75% and 0.5% baths (figures 6(C) and (D)). This is in strong agreement with the findings from the hanging drop experiments, in that greater fusion was seen in baths of lower stiffness. This can be attributed to lower stiffness of the baths, so the filaments were not stabilised as much as the as-made baths. After 7 d, these differences were less marked. Similar trends were seen in the surface roughness measurements with a significant reduction in Rq of the 0.5% bath after 48 h (figures 6(F) and (G)). Previous work on chondrocyte spheroids in the formation of micro and macro tissues has shown that without support materials, roughly spherical tissues are formed after 7 d (40), indicating that we have provided sufficient geometrical restraint on the tissues to ensure that the printed structures are maintained for 7 d. While individual spheroid 3D reconstructions were visualised previously, the tissue clearing process used is not sufficient to image these printed structures within agarose suspension baths due to the granular nature of the bath causing refraction and limiting light penetration through multi-spheroid prints.

Once basic characterisation of printed lines was evaluated, more complex 3D printed shapes were achieved with spheroid-only inks, including 6 mm cylinders printed within 0.75% ‘as made’ suspension baths (figure 6(H)). The suspension media was sufficiently stiff to prevent compaction and balling of the spheroids with culture. However, some gaps emerged between printed lines during culture, likely due to irregular fusion and displacement of the agarose gel during printing (figure 6(H)).

**Figure 7.**
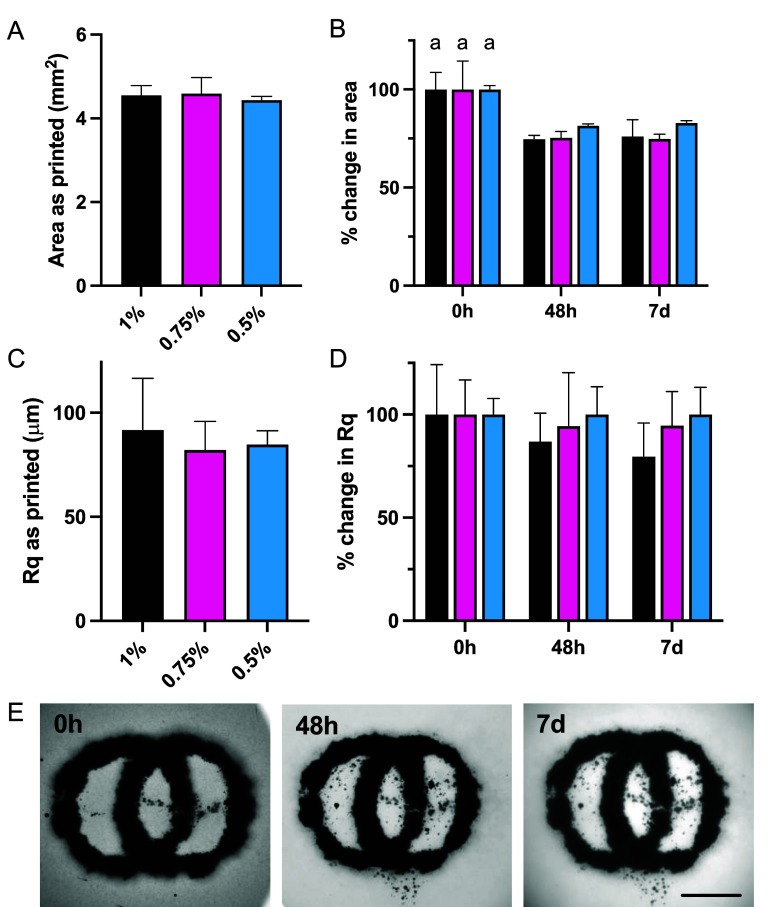
Printing of MSC spheroid-only inks into agarose suspension baths. Area of filaments (A) ‘as printed’ and (B) over time in ‘as made’ suspension baths. Rq of filaments (C) ‘as printed’ and (D) over time in ‘as made’ suspension baths. (E) Complex overlapping rings to determine effects of fusion and compaction in larger structures (Scale Bar: 3 mm). *n* = 4–12 lines per formulation for quantification.

To ensure applicability across multiple cell types, MSC spheroids were also investigated in ‘as made’ baths (figure 7). Similar compaction was observed between printing and 48 h of culture in the suspension media with 20%–25% reduction in area (figure 7(B)) compared to 25%–30% in the chondrocyte spheroids (figure 6(C)). The change in surface roughness (Rq) of the filaments was less marked in MSCs with decreases, albeit not statistically significant, observed in 1% baths after 48 h and 7 d (figure 7(D)). Overlapping structures were very stable over 7 d with good fusion but no breakup or unintended fusion of printed regions (figure 7(E)). Printed MSC constructs could undergo chondrogenesis in the presence of the appropriate soluble factors in the media, or other cell types based on the biochemical and biophysical signals provided.

**Figure 8.**
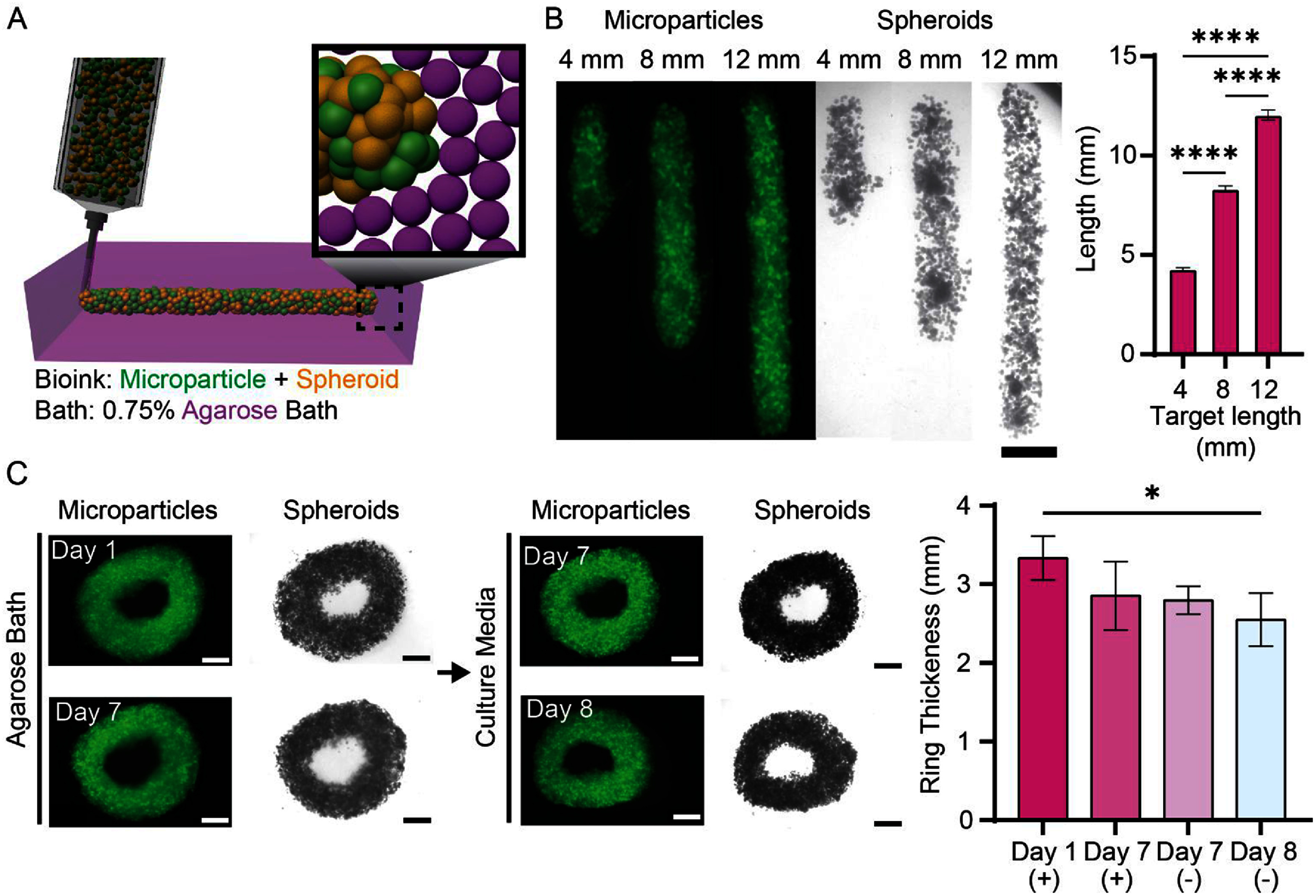
Printing of MSC spheroid-microgel composite inks into agarose suspension baths. (A) Schematic of the printing of a mixture of spheroids and microgels into an agarose suspension bath. (B) Representative examples of NorHA (grey) microgel and spheroid (brightfield) printed filaments of various lengths within an 0.75% agarose suspension bath (Scale Bar: 2 mm). (C) Representative fluorescent and brightfield images of 6 mm rings of spheroid and microparticle inks printed in 0.75% agarose and cultured for 7 d (left). Agarose suspension bath was removed on day 7 and cultured for an additional day to demonstrate granular composite stability. Scale Bar: 2 mm. Quantification of ring thickness throughout the culture period in agarose (+) or in culture media (−) (right). *n* = 4. *P*: *⩽ 0.05.

To validate further the use of spheroid-based bioinks, spheroid and hydrogel microparticle bioinks comprised of MSC spheroids and norbornene-modified hyaluronic acid microparticles of similar size were formed and printed into lines and rings within agarose baths (figure 8(A)). Hydrogel microparticles add to the potential complexity of the printed materials, as they can be chemically crosslinked together for stability or be used to deliver various soluble factors, such as growth factors. The mixtures of spheroids and microgels were printable within the bath, selected as 0.75% based on the work above (figure 8(B)). The printed lines matched the desired input lengths as well. It should be noted that the concentration of the microgels can influence the print properties—if there are too many microparticles this can interfere with spheroid fusion, especially in thin lines where there may be regions without spheroids across the entire line. Rings were also printed with these mixtures in the agarose bath, which allowed spheroid–spheroid fusion over time, maintaining the integrity of the ring structure over 7 d (figure 8(C)). We also showed that these rings could be removed from the bath and cultured an additional day in media and retained their structure. This approach has a lot of potential to leverage the benefits of spheroid inks and their fusion into tissues with microparticles that can impart added functionality.

## 4. Conclusions and limitations

This study investigates the bioprinting of a spheroid-only bioink that can be printed, fused and cultured in an agarose fluid gel suspension bath without requiring additional stabilisation. The findings show that lower polymer concentrations in the suspension bath promote faster spheroid fusion, which is critical for the formation of cohesive tissue structures. However, initial print fidelity and stability over culture is challenging when the suspension bath is too fluid. These insights will be advantageous for the optimisation of suspension baths for spheroid-based bioprinting, potentially enabling more effective tissue engineering applications that better mimic natural developmental processes. Ultimately, we defined a range of agarose fluid gel suspension baths that supported the printing and culture of spheroid-based bioinks, including those that are formed simply from the jamming of cell spheroids or those fabricated with hydrogel microparticles present which could expand the potential of printed structures further.

Despite these exciting findings, there are limitations to the work as reported. For example, we have focused on only one class of suspension baths, although there are wide classes of suspension baths now used within the field. This specific bath was selected for simplicity in use, ability to modify with CM for extended culture, and potential for spheroid fusion, but other baths could also be investigated with this approach. We also selected relatively simple patterns of lines and rings as proof of concept and relatively short-term cultures that we believe advance the platform. However, towards tissue engineering, larger and even more complex structures could be investigated. From the results of this study, we can define a key criterion for the bioprinting of spheroid-only bioinks. Minimum support bath moduli are required to ensure that the force of spheroid fusion does not exceed the restraint of the bath. If this modulus is not met, printed filaments will ball or fracture at imperfections in the print.

## Data Availability

The data that support the findings of this study are available as supplementary material.
